# Long-term effectiveness of tocilizumab in patients with rheumatoid arthritis, stratified by number of previous treatment failures with biologic agents: results from the German RABBIT cohort

**DOI:** 10.1007/s00296-017-3870-7

**Published:** 2017-11-16

**Authors:** Lisa Baganz, Adrian Richter, Jörn Kekow, Arnold Bussmann, Andreas Krause, Carsten Stille, Joachim Listing, Angela Zink, Anja Strangfeld

**Affiliations:** 1 Epidemiology Unit, German Rheumatism Research Centre, Charitéplatz 1, 10117 Berlin, Germany; 2 Otto-von-Guericke University, Magdeburg, Germany; 3 Geilenkirchen, Germany; 4 Berlin, Germany; 5 Hannover, Germany; 6 Charité University Medicine Berlin, Berlin, Germany

**Keywords:** Observational cohort study, Treatment strategy, Biologics register, IL-6 blockade, Line of therapy

## Abstract

In Germany, Tocilizumab (TCZ) is used for the treatment of rheumatoid arthritis both in biologic-naïve patients and those with previous failures of biologic disease-modifying antirheumatic drugs (bDMARDs). The long-term effectiveness and retention rates of TCZ in patients with different numbers of prior bDMARD failures has rarely been investigated. We included 885 RA patients in the analyses, enrolled with the start of TCZ between 2009 and 2015 in the German biologics register RABBIT. Patients were stratified according to prior bDMARD failures: no prior bDMARD or 1, 2 or ≥ 3 bDMARD failures. We applied Kaplan–Meier methods and Cox-regression to examine treatment adherence as well as linear mixed effects models to investigate effectiveness over 3 years of follow-up. Compared to biologic-naïve patients, those with prior bDMARD failures at start of TCZ were younger but had significantly longer disease duration and more comorbidities. DAS28 at baseline and loss of physical function were highest in patients with ≥ 3 bDMARD failures. During follow-up, patients with up to two bDMARD failures on average reached low disease activity (LDA, DAS28 < 3.2). Those with ≥ 3 prior bDMARDs had a slightly lower response. However, after 3 years, nearly 50% of them achieved LDA. Treatment continuation on TCZ therapy was similar in patients with ≤ 2 bDMARD failures but significantly lower in those with ≥ 3 bDMARD failures. TCZ seems to be similarly effective in patients with no, one or two prior bDMARD failures. The majority of patients achieved LDA already after 6 months and maintained it over a period of 3 years. TCZ proved effective even in the high-risk group of patients with more than two prior bDMARD failures.

## Introduction

With the advent of biologic disease-modifying antirheumatic drugs (bDMARDs), the treatment options for the management of rheumatoid arthritis (RA) have improved tremendously. The availability of treatments with different mechanisms of action addresses the need for therapies targeting different components of the immune system since patients may not respond to a specific bDMARD therapy but to another mode of action. Since 2013, the EULAR guidelines entitle remission or low disease activity (LDA) to be the primary treatment target in all RA patients [1]. If the target cannot be reached within 6 months after initiating treatment, the therapy should be altered by adding conventional synthetic (cs)DMARDs, switching to a bDMARD and consequently also between bDMARDs. Expedited therapy decisions involve sequential use of different DMARDs. Therefore, more recently licensedbDMARDs representing new modes of action are often prescribed as second-, third- or fourth-line therapies after failure of a tumor necrosis factor α inhibitor (TNFi). This practice reflects limited long-term data about safety and effectiveness of new drugs rather than evidence of effectiveness after bDMARD failure.

Determining an optimal treatment algorithm for RA patients is methodologically complex. Various studies compared the effectiveness of switching to a second TNFi or to a bDMARD with another mode of action after one TNFi failure [2–5]. The studies showed that in patients who switched to a bDMARD with a different mode of action the drug survival [2, 3], improvement in DAS28 (Disease Activity Score based on 28 joint counts) [5] and the remission rates [4] were higher [2–5]. Other studies showed that a second TNFi can be effective, specifically after discontinuation because of adverse events [6, 7], and no differences in outcomes (CDAI, ACR responses, mHAQ) were found for patients with prior TNFi exposure who received either another TNFi or abatacept [8]. It has been recommended to use different mechanisms of action at least after the second TNFi-failure [9, 10]. Heretofore, no clear recommendation about the optimal sequence of different DMARDs is available [1].

TCZ is a humanised monoclonal antibody targeting the IL-6 receptor. This bDMARD has been approved in 2009 for the treatment of RA and is administered either intravenously or subcutaneously (since 2014). Recommended doses of TCZ are 8 mg/kg once every 4 weeks (intravenously) or 162 mg every week irrespective of the patient’s weight (subcutaneously). Studies show that besides a higher incidence of injection side reactions, the safety and efficacy of subcutaneous TCZ are similar to intravenous treatment [11, 12] and also switching from intravenous to subcutaneous injection seems to have no influence on safety and efficacy [13, 14]. While TCZ is approved both as monotherapy and in combination with methotrexate (MTX), combinations with other csDMARDs are not. The recommendation is to use TCZ in combination with MTX [1]. The usage of TCZ as second line bDMARD after failure of TNFi which was shown to be an effective treatment option by several RCTs [15–17] is common in Germany. A recent study showed that in short-term use (6-months follow-up) TCZ was equally effective in biologic-naïve patients and patients with prior bDMARD failures [18]. However, it is not clear if in the long run response to treatment with TCZ is lower in patients with prior bDMARD failure compared to biologic-naïve patients. Furthermore, outcome and safety of TCZ treatment in patients with more than two bDMARD failures have been insufficiently investigated.

The aim of this study was to examine the effectiveness of TCZ over 3 years of follow-up depending on the patients’ prior exposure to bDMARDs. Due to the observational design of this study differences of patients’ baseline characteristics are taken into account as well as concomitant treatment with csDMARDs and time-varying doses of glucocorticoids (GCs). Furthermore, the impact of attrition in this cohort of RA patients is examined as well as reasons for discontinuing treatment with TCZ.

## Subjects and methods

### Data source

We analysed data from the German biologics register RABBIT (Rheumatoid Arthritis: Observation of Biologic Therapy) which is an ongoing observational cohort study initiated in 2001. RA patients can be included if they start a licensed bDMARD, biosimilar or a csDMARD therapy after at least one csDMARD failure. At baseline, after 3, 6 and then every 6 months, rheumatologists report start and stop dates of actual DMARD therapies, prior DMARD therapies, glucocorticoid doses, comorbidities (at baseline, after 2.5, 5 and 7.5 years), adverse events and features of the clinical status such as the disease activity measured by DAS28 and its components [including erythrocyte sedimentation rate (ESR)]. Patient-reported outcomes are recorded at the same visits. Physical function is captured by a German instrument [Funktionsfragebogen Hannover (FFbH)] similar to the Health Assessment Questionnaire [19]. If patients stop their baseline therapy, they are not excluded but they will be observed for up to 10 years regardless of their treatment.

If patients have not visited the rheumatologist for more than 1 year without information, extensive dropout investigations are carried out including enquiries to doctors, patients themselves or their relatives. Vital status and causes of death are requested from the local administration and health offices. Further details of design and conduct of the RABBIT study were reported elsewhere [20].

### Patient selection

The enrolment of patients initiating TCZ treatment started in 2009. Until 31 October 2015, 950 patients were enrolled into RABBIT. We excluded 65 patients from the analysis for whom currently only baseline data were available. In total, 885 patients contributed to the analyses. Patients who missed at least two scheduled visits were considered as dropouts.

### Statistical analysis

Patients were stratified into four groups according to the number of bDMARD failures prior to initiation of TCZ. We compared baseline characteristics of patients with one or more bDMARD failures with those of biologic-naïve patients (reference group).

Therapy discontinuation within 36 months of follow-up was examined using Kaplan–Meier methods; Cox-proportional hazard models were applied to compare retention rates between strata of bDMARD failures. We defined discontinuation as the end of the first TCZ therapy after the enrolment in RABBIT. In addition, we investigated the time to stop TCZ with different concomitant csDMARD therapies.

The effectiveness of TCZ was evaluated using the DAS28-ESR over the first 3 years after treatment initiation. We applied two different linear mixed models: the first model is a completer analysis including only patients who maintained TCZ treatment throughout their complete follow-up. In the second model, we considered all patients initiating TCZ treatment at baseline (intent-to-treat (ITT) approach). Since the dropout processes as well as the numbers of patients who switched to another bDMARD were not equally distributed between the strata, we applied multiple imputations for the DAS28 for those patients who switched to another bDMARD (stopped TCZ therapy) during follow-up or were lost to follow-up. The imputation model included age, number of comorbidities (0, 1, 2, ≥ 3), rheumatoid factor, number of prior bDMARD failures (0, 1, 2, ≥ 3), DAS28 and FFbH scores prior to the start of another bDMARD or dropout. Both linear mixed models were adjusted for the following baseline covariates: disease duration, functional status, DAS28, number of comorbidities (0, 1, 2, ≥ 3), number of prior bDMARD failures (0, 1, 2, ≥ 3). Additionally, we tested for a different course of the disease activity (DAS28) between strata of bDMARD failures by an interaction term of follow-up time and the number of bDMARD failures. In a subanalysis, we included the dose of TCZ (≤ 6 vs. > 6 mg/kg) in the model. To examine whether the use of concomitant csDMARDs had an influence on the effectiveness, we included a time-varying categorical variable for csDMARD therapy in the linear mixed model with imputed data for the DAS28. We distinguished between TCZ monotherapy, TCZ + MTX and TCZ + other csDMARD.

In addition, we applied generalized linear mixed models (glimmix procedure in SAS 9.4) to calculate mean predicted probabilities for (a) low disease activity, (b) high disease activity and (c) ≤ 2 swollen joints. In model (a) and (b), we used the same covariates for adjustment as in the analyses of effectiveness (DAS28). In model (c) we additionally adjusted for baseline SJC. Instead of multiple imputation techniques for missing values of the swollen joint counts, we used last-observation-carried-forward (LOCF) since the joint counts follow a highly skewed distribution with a concentration on zeros, i.e., imputation of negative values are likely. The same approach (LOCF) was used for missing doses of concomitant glucocorticoids. In a linear mixed model, we investigated whether the glucocorticoid doses differed between the strata during follow-up. We adjusted for time (discrete follow-up visits), number of prior bDMARD failures (0, 1, 2, ≥ 3) and the interaction of time and bDMARD failures. For analysis, we used software from the SAS Institute, version 9.4.

## Results

### Baseline characteristics

Most of the 885 patients enrolled with TCZ (64.1%) had one or more bDMARD failures (Table 1): 318 (35.9%) were biologic-naïve (first-line TCZ), 286 (32.3%) had one bDMARD failure (second-line TCZ), 186 (21.0%) two (third-line TCZ), and 95 (10.7%) ≥ 3 prior bDMARD failures (fourth-line TCZ). Patients with prior bDMARD failures were significantly different from biologic-naïve patients: they were younger, had longer disease duration, more csDMARD failures, more erosive changes and more severe fatigue. The functional status was significantly lower in patients with one or at least three bDMARD failures and more patients had three or more comorbidities compared to biologic-naïve patients. In the group with ≥ 3 prior bDMARD failures, nearly 50% of the patients had three or more comorbidities, in particular significantly more osteoporosis, diabetes and heart failure than biologic-naïve patients. No difference was found for fibromyalgia, psoriasis and depression.

**Table 1:** Baseline characteristics of patients enrolled with tocilizumab

	Tocilizumab as			
	1st line	2nd line	3rd line	4th line
Number of patients	318	286	186	95
Female patients, *n* (%)	239 (75.2)	227 (79.4)	149 (80.1)	77 (81.1)
Age	58 ± 12.5	56.4 ± 12.4	55.7 ± 12.7	54.6 ± 14.8*
Disease duration in years	8 ± 7.4	11.6 ± 8.5*	13.3 ± 9.2*	15.3 ± 9.9*
TCZ dose mg/kg	7.1 (2.1)	7.3 (2.0)	7.4 (1.8)	7.2 (2.2)
TCZ dose ≤ 6 mg/kg, *n* (%)	54 (17.1)	38 (13.5)	20 (10.9)	14 (15.2)
Prev. TNFi, *n* (%)	0	262 (91.6)	185 (99.5)	95 (100)
Prev. other bDMARDs, *n* (%)	0	24 (8.4)	24 (15.1)	57 (64.2)
Concomitant csDMARD, *n* (%)	170 (53.5)	141 (49.3)	97 (52.2)	47 (49.5)
There of MTX only, *n* (%)	102 (60.0)	96 (68.09)	69 (71.13)	35 (74.47)
GC dose mg/d	8.2 ± 5.1	7.5 ± 4.0	8.9 ± 8.6	7.8 ± 4.3
No GCs, *n* (%)	121 (38.1)	103 (36)	63 (33.9)	29 (30.5)
DAS28	5.1 ± 1.3	5.2 ± 1.3	5.2 ± 1.3	5.5 ± 1.3*
ESR in mm/h	34.2 ± 26.2	31.1 ± 23	33.5 ± 25.6	36.5 ± 25.9
CRP in mg/L	16.5 ± 22	17.9 ± 26.5	19.8 ± 32.1	20.7 ± 24
SJC	6.7 ± 5.2	6.5 ± 5.1	6.2 ± 5.3	7.3 ± 6.6
TJC	8.5 ± 6.5	9.1 ± 6.9	8.7 ± 6.8	10.2 ± 7.8*
ACPA pos., *n* (%)	165 (68.5)	151 (72.9)	104 (73.8)	52 (73.2)
RF pos., *n* (%)	217 (71.9)	208 (75.1)	132 (72.9)	64 (68.1)
Erosive RA, n (%)	164 (53.9)	188 (68.1)*	110 (64.3)*	66 (75)*
FFbH	65.8 ± 23	60.5 ± 24.5*	62.1 ± 23.6	56.1 ± 23.9*
Pain (NRS: 0–10)	5.9 ± 2.3	6.3 ± 2.2*	6.2 ± 2.1	6.8 ± 1.9*
PGA (NRS: 0–10)	5.9 ± 2.1	6.2 ± 2	6.2 ± 1.9	6.7 ± 1.9*
Fatigue (NRS: 0–10)	5 ± 2.9	5.5 ± 2.8*	5.6 ± 2.5*	6.3 ± 2.4*
Comorbidities				
Osteoporosis, *n* (%)	53 (16.7)	64 (22.4)	29 (15.6)	28 (29.5)*
Diabetes, *n* (%)	33 (10.4)	34 (11.9)	14 (7.5)	22 (23.2)*
Heart failure, *n* (%)	10 (3.1)	5 (1.7)	4 (2.2)	8 (8.4)*
Hypertension, *n* (%)	134 (42.1)	122 (42.7)	67 (36)	37 (38.9)
1 comorbidity, *n* (%)	83 (26.1)	65 (22.7)	59 (31.7)	14 (14.7)
2 comorbidities, *n* (%)	57 (17.9)	50 (17.5)	35 (18.8)	16 (16.8)
≥ 3 comorbidities, *n* (%)	96 (30.2)	111 (38.8)*	51 (27.4)	45 (47.4)*

Values are mean ± SD unless otherwise specified

*No* number, *SD* standard deviation, *prev* previous, *TNFi* tumor necrosis factor inhibitor, *bDMARDs* biologic disease modifying antirheumatic drugs, *csDMARDs* conventional synthetic disease modifying antirheumatic drugs, *GC* glucocorticoid, *DAS28* disease activity score based on 28 joints, *ESR* erythrocyte sedimentation rate, *CRP* C-reactive protein, *SJC* swollen joint counts, *TJC* tender joint counts, *ACPA*, citrullinated peptide/protein antibodies, *pos* positive, *RF* rheumatoid factor, *FFbH* Funktionsfragebogen Hannover, *NRS* numerical rating scale, *PGA* patient global assessment

* Significantly different from patients with first line TCZ (*p* < 0.05)

#### Patient follow-up

Overall, 379 patients (first-line: 118, second-line: 119, third-line: 83, fourth-line: 59) terminated TCZ but were still under observation in the register with other DMARD treatments. In contrast, only 60 out of 885 patients (6.8%) were lost to follow-up over 3 years of observation (first-line: 19, second-line: 17, third-line: 21, 4th-line: 3). Apart from discontinuation and dropout, a total of 288 patients (1st-line: 120, 2nd-line: 87, 3rd-line: 63, fourth-line: 18) were enrolled later than 3 years prior to October 31 2015 and could therefore not complete 3 years of follow-up.

### Therapy adherence

#### Retention rates

**Fig. 1:**
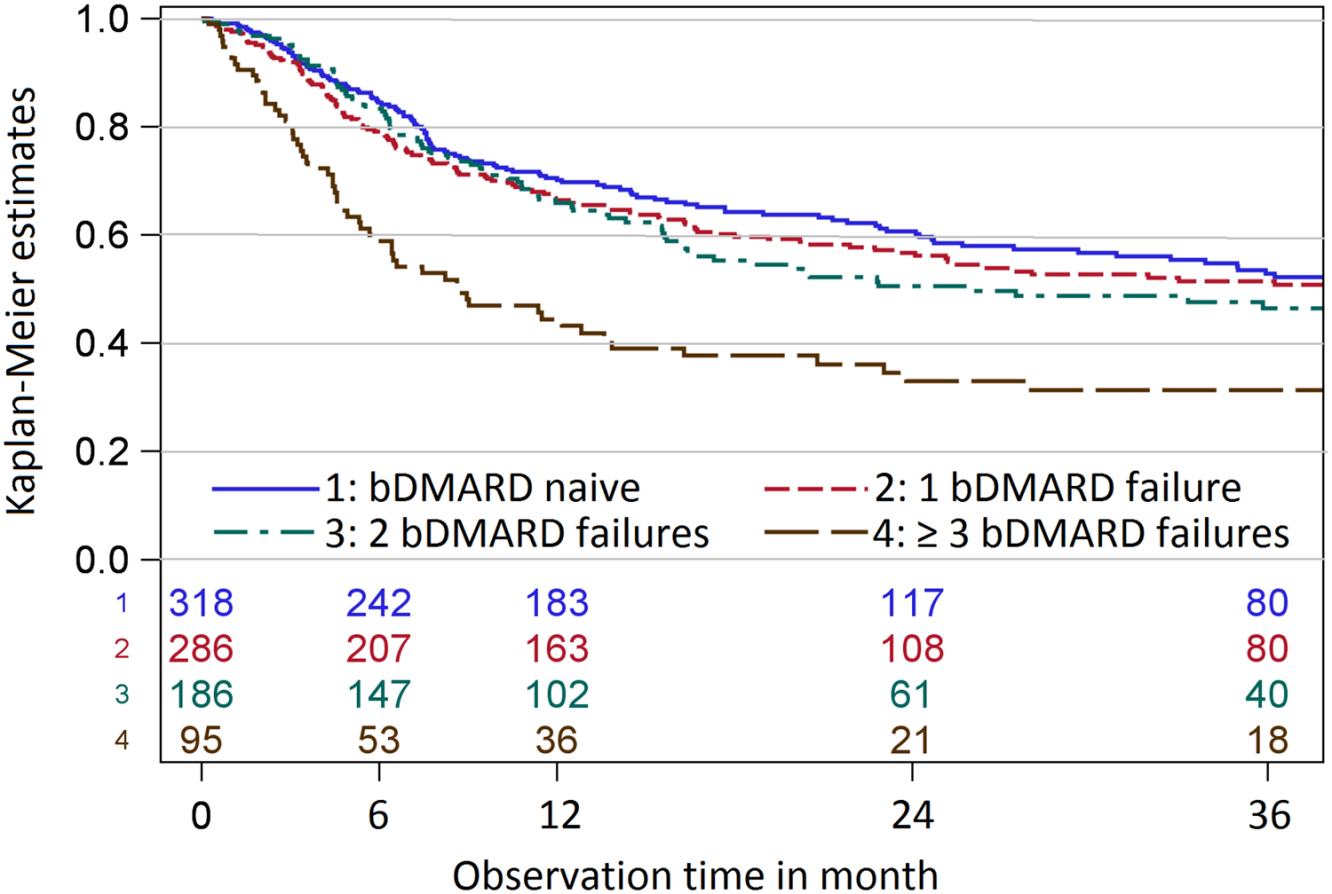
Kaplan–Meier curves for retention of tocilizumab therapy over 3 years. The patients were stratified by the number of prior bDMARD failures. The numbers on top of the x-axis represent the number of patients at risk at the corresponding time point

After the first year, 65–70% of the patients with ≤ 2 prior bDMARD failures were still on TCZ therapy compared to only 43% in the group with ≥ 3 bDMARD failures (Fig. 1). The Kaplan–Meier estimates after 3 years of follow-up were also similar in the first three strata: 52.2% (bDMARD naïve), 50.8% (1 bDMARD failure) and 46.5% (2 bDMARD failures) compared to 31.3% in the group with ≥ 3 bDMARD failures. In the group with three or more bDMARD failures, 41% of patients stopped TCZ therapy already during the first 6 months.

#### Characteristics of patients discontinuing TCZ

The unadjusted hazard ratios show that the probability for stopping TCZ therapy was 2.2 times higher in patients with at least three prior bDMARD failures compared to biologic-naïve patients and 1.8 times higher compared to patients with two prior bDMARD failures.

Patients who discontinued TCZ had higher doses of TCZ at baseline (> 6 mg/kg) than those who maintained their treatment. They also had higher DAS28 at baseline and more often three or more comorbidities, especially diabetes. Besides biologic-naive patients, those who discontinued had a lower functional status.

#### Reasons for terminations

The most frequent reasons for discontinuation were adverse events and ineffectiveness (multiple reasons could be named). In biologic-naïve patients who discontinued TCZ treatment, 32% stopped because of ineffectiveness and 47% due to adverse events (AEs). After one failure (2 failures, ≥ 3 failures), 38% (46, 42%) stopped due to ineffectiveness and 57% (47, 53%) due to AEs. During the first 6 months the percentage of patients who stopped TCZ due to AEs was 15% (17, 35%) higher for patients with no (1; ≥ 3) prior failures than during the following 2.5 years. Overall, the most frequent serious adverse event was “hospitalized surgery”. In patients with ≥ 3 bDMARD failures, this event occurred 14.9 times per 100 patient-years, whereas the incidence rates in the other three groups were 3–7 per 100 patient-years. More than 75% of the surgeries were bone and joint surgeries in all strata. In patients who stopped TCZ therapy, the rates for serious infections were 36.5 (first-line), 33.4 (second), 48.5 (third), and 49.2 (fourth) per 1000 py, and rates for neoplasms were 14.6 (first-line), 20.0 (second), 19.4 (third), and 0 (fourth). This was higher than in patients who continued therapy (serious infections: IR: 7.8–27.3; neoplasms: IR: 4.8 in third-line and no event in all other patients). There was no difference regarding the occurrence of major cardiovascular events. Four patients died under TCZ therapy.

### Effectiveness

In both approaches, all patients had significant DAS28 improvements, irrespective of the number of prior bDMARD failures. In the first approach, only 335 patients who remained on TCZ were included (completers). Of them, 130 (39%) were biologic-naïve, 111 (33%) had one bDMARD failure, 70 (21%) had two and 24 (7%) had three or more prior failures. The percentage of completers after 3 years of follow-up was 41% in biologic-naive patients, 39% in second line, 38% in third line and 25% in fourth line users. The average improvement after 3 years of follow-up varied between 2.4 and 3.0 DAS28 units. On average, the patients reached LDA (Table 2).

**Table 2:** Adjusted baseline DAS28 and least-square means of the course of the DAS28 over 3 years of follow-up separated by the number of prior bDMARD-failures

	*N*	Baseline	Month 6	Month 12	Month 24	Month 36
Model 1: Completers						
1st-line	130	5.2	2.42 [2.18;2.66]	2.51 [2.26;2.77]	2.26 [1.97;2.55]	2.23 [1.91;2.55]
2nd-line	111	5.2	2.81 [2.55;3.07]	2.82 [2.56;3.09]	2.74 [2.45;3.03]	2.55 [2.24;2.87]
3rd-line	70	5.2	2.96 [2.63;3.28]	2.84 [2.49;3.18]	2.64 [2.27;3.01]	2.61 [2.18;3.03]
4th-line	24	5.2	2.62 [2.04;3.19]	2.68 [2.07;3.28]	2.81 [2.10;3.51]	2.87 [2.14;3.60]
Model 2: ITT						
1st-line	318	5.2	2.79 [2.62;2.96]	2.84 [2.66;3.02]	2.84 [2.65;3.02]	2.50 [2.25;2.74]
2nd-line	286	5.2	3.00 [2.82;3.17]	3.06 [2.86;3.25]	2.97 [2.78;3.15]	2.86 [2.64;3.07]
3rd-line	186	5.2	3.08 [2.85;3.30]	3.06 [2.83;3.29]	3.04 [2.76;3.32]	2.70 [2.39;3.00]
4th-line	95	5.2	3.48 [3.18;3.78]	3.55 [3.22;3.87]	3.14 [2.72;3.56]	3.27 [2.83;3.70]

We adjusted for the following baseline variables: disease duration, functional status, DAS28 and the number of comorbidities

In approach 2 (ITT analysis), the DAS28 was reduced by 2.0–2.7 points on average after 3 years. Consequently, the estimated means of the DAS28 were higher than in the first model. After 36 months, patients with less than three bDMARD failures on average reached LDA, whereas patients with ≥ 3 bDMARD failures remained on average in moderate disease activity. The difference in the DAS28 scores after 3 years between biologic-naïve patients and those with three or more prior bDMARD failures is larger than in the completer analysis but still not significant (Table 2). Additional adjustment for the dose had no influence on the results. However, the overall mean of the DAS28 averaged over 3 years was significantly higher in patients with ≥ 3 prior bDMARD failures compared to biologic-naïve patients or patients with 1 bDMARD failure [first-line: 2.79 (2.67;2.91), second-line: 2.96 (2.84;3.09), third-line: 3.00 (2.84;3.15), fourth-line: 3.34 (3.10;3.59)]. This result is supported by the predicted probability for achieving LDA over 3 years. It was decreasing from 66 to 48% with the number of prior bDMARD failures. A similar result was also obtained for swollen joint counts (SJC): the mean probability over 3 years for ≤ 2 SJC decreased from 74 to 62% (Table 3). The probability for remaining in high disease activity was 10% in patients with ≥ 3 bDMARD failures.

**Table 3:** Mean baseline SJC and adjusted least-square means of the percentage of patients with SJC ≤ 2 for all strata of prior bDMARD failures

	*N*	Baseline	Month 6	Month 12	Month 24	Month 36
1st-line	318	6.6	75 [70;80]	80 [76;85]	76 [71;82]	78 [72; 84]
2nd-line	286	6.6	65 [59;71]	67 [61;73]	66 [60;72]	69 [62; 76]
3rd-line	186	6.6	64 [56;71]	67 [59;74]	66 [58;74]	66 [58; 75]
4th-line	95	6.6	59 [48;70]	60 [49;71]	70 [59; 81]	68 [56;80]

We adjusted for the following baseline variables: disease duration, functional status, DAS28, SJC and the number of comorbidities

*ITT* intention-to-treat

### Concomitant therapy

#### Conventional synthetic DMARDs

Overall, 430 patients (first-line: 148, second-line: 145, third-line: 89, fourth-line: 48) started TCZ as monotherapy. Of them, 73 (17%, first-line: 12%, second-line: 20%, third-line: 16%, fourth-line: 25%) added a csDMARD during observation. There was no difference in effectiveness between TCZ monotherapy or TCZ in combination with MTX or another csDMARD or more than one csDMARD. Compared to concomitant MTX only use, TCZ monotherapy resulted on average in an insignificantly higher DAS28 of 0.03 [− 0.1; 0.2] points and the effect of other combinations was 0.04 [− 0.1; 0.2]. Moreover, the time to stop TCZ therapy was similar for the three groups (data not shown).

#### Glucocorticoids

Overall, 9.6% [95%-KI:(7.7; 11.7%)] of patients were enrolled with a high GC dose (> 10 mg/d) and 38.3% did not receive GCs at baseline. However, the mean GC doses during follow-up barely differed between the strata: 3.1 (first-line), 3.4 (second-line), 4.1 (third-line), and 4.1 mg/d (fourth-line).

**Fig. 2:**
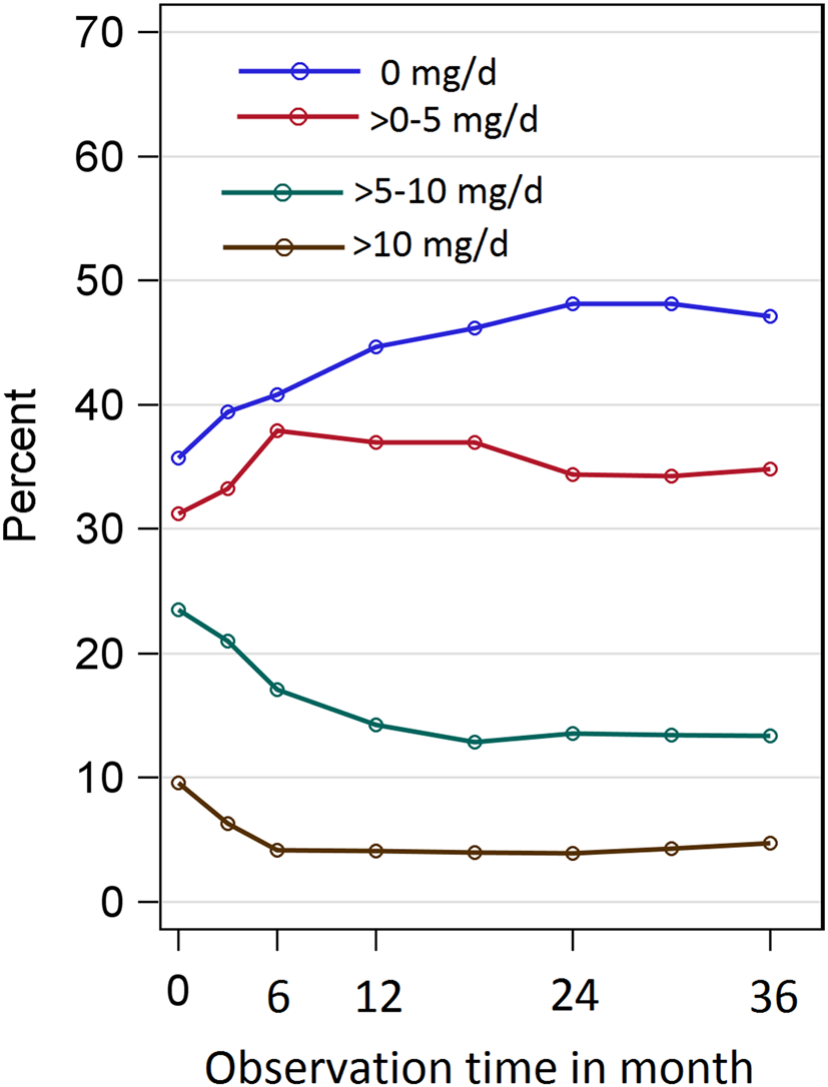
Time-varying percentage of patients who received either no concomitant glucocorticoids, > 0–5, > 5–10  or > 10 mg/d. Missing glucocorticoid doses and doses of patients who switched to another biologic where imputed using LOCF

During follow-up, the percentage of patients with high GC doses decreased and the percentage of patients without GCs increased (Fig. 2). The changes were larger for the patients who were enrolled later.

## Discussion

We investigated the effectiveness of TCZ in 885 RA patients over 3 years stratified by the number of prior bDMARD failures incorporating third- and fourth-line bDMARD therapies. To our knowledge, there is little data on this issue. However, due to the chronicity of RA and an increasing number of patients with numerous treatment failures, more analyses of this kind are necessary.

Our results show that TCZ is an effective treatment in the majority of biologic-naïve patients as well as in those with up to three prior bDMARD failures. Particularly, TCZ completers achieved an average disease activity corresponding to LDA. However, after adjustment for therapy switches and dropout processes (ITT analysis), we did also find no major differences between strata: the mean DAS28 was reduced by at least 2 units and on average LDA was reached in patients with ≤ 2 bDMARD failures. Similar results have been reported by Harrold et al. regarding the median decrease in CDAI, improvement in mHAQ and mACR20/50/70 responses [21] and Kihara et al. who also found no differences in EULAR response and DAS28-remission at month 6 for TCZ-treated patients being either biologic-naïve or after at least one bDMARD failure [18].

In our analysis, merely the patient group with ≥ 3 bDMARD failures had a slightly lower response in the ITT approach. This result is in line with RCT data showing a similar ACR20 response in patients with one, two or three prior TNFi-failures but lower ACR50 and ACR70 responses for patients with ≥ 3 TNFi failures [16]. It is likely that the latter patients form a risk group for non-response and adverse events irrespective of the therapy [22]. This was also apparent in our data: although patients with ≥ 3 prior bDMARDs were younger, they had significantly longer disease duration, nearly two-thirds had already been exposed to non-TNFi prior to TCZ, and about 50% had three or more comorbidities. In addition, these patients developed more SAEs involving especially surgeries (there of 75% bone and joint-related surgeries) with an incidence of 14.9 per 100 patients-years which was more than twice as high as in the other groups. However, the disease-related bone and joint surgeries are very likely not associated with the recently initiated TCZ treatment. They will rather be a consequence of a long-standing and insufficiently controlled disease. A similar observation was made in the study of Kihara et al., wherein patients with subsequent-line TCZ had more often a history of joint replacement, longer disease duration, lower physical function and more comorbidities [18]. On the other hand, our study also shows that in a considerable proportion of patients with ≥ 3, bDMARD failure disease activity (DAS28) was significantly reduced; in the ITT analysis, 48% of these patients achieved LDA. Overall, the results on effectiveness and incidence of SAEs are summarized by our time-to-event analysis. Assuming that time-to-event is a combined surrogate marker for effectiveness and safety, we found similar survival curves for patients with 0, 1 or 2 bDMARD failures and a significantly lower retention rate for patients with ≥ 3 bDMARD failures.

Our results are not compromised by a differential use of concomitant GCs or csDMARDs. The impact of concomitant methotrexate or other csDMARDs was marginal and insignificant compared to TCZ monotherapy. This is in contrast to the EULAR guidelines recommending TCZ in combination with MTX [1] but supported by the study of Kihara et al., wherein concomitant MTX therapy was not associated with a better response to TCZ. Regarding concomitant GCs, we could not find systematically different doses between strata. In all strata, doses of concomitant GCs were significantly reduced during follow-up. This is in agreement with two recent studies [23, 24]. In patients enrolled after 2012, more than 50% completely withdrew GCs under TCZ treatment. This is in line with the EULAR recommendations to withdraw concomitant GCs as soon as possible [1].

This study has limitations inherent to the observational design. We observed different dropout rates across strata of bDMARD failures which may have led to biased estimates. Therefore, we considered missing values and dropouts by multiple imputations. An imputation model will not nullify the impact of non-random dropouts but the comparison of ITT (model 2) versus completer analyses (model 1) showed an expected upwards correction of values of the DAS28 over time in model 2. In addition, the overall low rates of missing values and the low attrition rate in RABBIT support the significance of the findings [20]. Due to the suppression of ESR by TCZ [25], the outcomes DAS28-ESR and LDA may overestimate the treatment effect. Our results should, therefore, not directly be compared to data on other substances. Nevertheless, comparisons of the DAS28-ESR among users of TCZ remain valid. In order to estimate treatment effectiveness without the impact of ESR, we analysed the percentage of patients achieving ≤ 2 swollen joints over 3 years which was between 74 (bio-naïve) and 62% (≥ 3 bDMARD failures) (data not shown). This underlines our results on clinical effectiveness.

Strengths of this study are the large number of patients receiving TCZ, enabling stratified analyses, and the follow-up of 3 years. Further, since all patients are from the same country, similar prescription guidelines and treatment patterns can be assumed. This is specifically important regarding the analysis of concomitant therapies since use and doses in daily practice may vary considerably between countries [26]. Moreover, regarding the use of glucocorticoids, the RABBIT study comprises not only doses at baseline but also during follow-up. Although the overall amount of missing data and dropouts was low, we implied imputation methods to control for this possible confounder.

## Conclusions

In conclusion, the results from this study indicate that TCZ is equally effective in patients with no, one or two previous bDMARD failures. The majority of patients achieved LDA and maintained it over a period of 3 years. In patients with more than two prior bDMARD failures overall effectiveness was lower, compared to the other patient groups. Nevertheless, nearly 50% of these patients reached LDA at follow-up. This is remarkable since these patients had a mean disease duration of 15 years, and a large number of previous joint surgeries, indicating a severe course of disease. Effective treatment options such as TCZ are needed for these difficult-to-treat patients.
